# *Cordyceps militaris* Fungus Extracts-Mediated Nanoemulsion for Improvement Antioxidant, Antimicrobial, and Anti-Inflammatory Activities

**DOI:** 10.3390/molecules25235733

**Published:** 2020-12-04

**Authors:** Esrat Jahan Rupa, Jin Feng Li, Muhammad Huzaifa Arif, Han Yaxi, Aditi Mitra Puja, Ahn Jong Chan, Van-An Hoang, Lalitha Kaliraj, Deok Chun Yang, Se Chan Kang

**Affiliations:** 1 Department of Oriental Medicinal Biotechnology, College of Life Sciences, Kyung Hee University, Yongin-si, Gyeonggi-do 17104, Korea; eshratrupa91@gmail.com (E.J.R.); jfh0109@naver.com (J.F.L.); Huzaifa_jutt@yahoo.com (M.H.A.); navycki@gmai.com (H.Y.); hoangan83@gmail.com (V.-A.H.); 2 Graduate School of Biotechnology, College of Life Sciences, Kyung Hee University, Yongin-si, Gyeonggi-do 17104, Korea; aditi@khu.ac.kr (A.M.P.); jongchanahn7@gmail.com (A.J.C.); lalithakr95@gmail.com (L.K.)

**Keywords:** *Cordyceps* extracts, *Cordyceps* nanoemulsion, ultra sonication, inflammation, antibacterial, antioxidant

## Abstract

This study aimed to produce and optimize a *Cordyceps militaris*-based oil-in-water (O/W) nanoemulsion (NE) encapsulated in sea buckthorn oil (SBT) using an ultrasonication process. Herein, a nonionic surfactant (Tween 80) and chitosan cosurfactant were used as emulsifying agents. The *Cordyceps* nanoemulsion (COR-NE) was characterized using Fourier-transform infrared spectroscopy (FT-IR), dynamic light scattering (DLS), and field-emission transmission electron microscope (FE-TEM). The DLS analyses revealed that the NE droplets were 87.0 ± 2.1 nm in diameter, with a PDI value of 0.089 ± 0.023, and zeta potential of −26.20 ± 2. The small size, low PDI, and stable zeta potential highlighted the excellent stability of the NE. The NE was tested for stability under different temperature (4 °C, 25 °C, and 60 °C) and storage conditions for 3 months where 4 °C did not affect the stability. Finally, *in vitro* cytotoxicity and anti-inflammatory activity were assessed. The results suggested that the NE was not toxic to RAW 264.7 or HaCaT (human keratinocyte) cell lines at up to 100 µL/mL. Anti-inflammatory activity in liposaccharides (LPS)-induced RAW 264.7 cells was evident at 50 µg/mL and showed inhibition of NO production and downregulation of pro-inflammatory gene expression. Further, the NE exhibited good antioxidant (2.96 ± 0.10 mg/mL) activity and inhibited *E. coli* and *S. aureus* bacterial growth. Overall, the COR-NE had greater efficacy than the free extract and added significant value for future biomedical and cosmetics applications.

## 1. Introduction

Medicinal plants have been used as therapeutic agents since ancient times, and more than 10,000 plants are used for medicinal purposes [1]. The medicinal value of plant depends on the active compounds found within them; however, natural active compounds often tend to be poorly absorbed, chemically unstable, and not particularly hydrophilic, and high doses and repeated administration may, therefore, be required to achieve therapeutic effects. A suitable delivery system is needed in order to improve the absorption of bioactive compounds from plant sources and thus to reduce the dose required and unwanted side effects. Nanoemulsions (NEs) can be effective carrier systems as therapeutic agents and have attracted attention in the food, cosmetic, and pharmaceutical industries. Recent studies have shown better solubility, permeability, and absorption of the active compound when formulated as an NE than as the free extract [2,3]. Nanoemulsions are submicron sizes emulsion ranging from 5 to 200 nm and are mostly developed using low- or high-energy methods comprising two immiscible phases with help of appropriate emulsifying agent [4]. Moreover, thermodynamic and kinetics both stability properties of nanoemulsion considered it as a safer drug carrier than other drug delivery system. Nanoemulsion formulations may be of the oil-in-water (O/W) or water-in-oil (W/O) type, depending on whether the water is dispersed on oil or *vice versa*. In O/W NE systems, oil and surfactant molecules are dispersed in an aqueous medium in which the surfactant acts to reduce the interfacial tension between the two immiscible liquids and make them fully miscible. The O/W NE type is more commonly used when preparing commercial products owing to its high kinetic stability (when formulated with nonionic surfactants and polymers), smaller droplet size, better lipophilic encapsulation components, ability to increase drug bioavailability of encapsulated active ingredients in an aqueous environment, and greater resistance to Ostwald ripening [5,6]. In this article, we focused on using ultrasonication to prepare an O/W NE combining a fungus extract, plant oil, nonionic surfactant (widely used in industry), and co-surfactant. An ultrasound method was adopted primarily because in this method, better encapsulation, higher yields, control of delivery, and smaller droplet size can be obtained using less energy than for any other conventional method [7]. An ultrasonic probe was used to produce a smaller NE compared with other ultrasonic baths. The O/W NE system we used consisted of a *Cordyceps* extract (COR-Ex), *Hippophae rhamnoides* (sea buckthorn) fruit oil, Tween 80 as surfactants, and chitosan from crab shells as a co-surfactant. *Cordyceps militaris*, widely known as parasitic fungus in China, has been extensively used as a traditional medicine. The active components of *Cordyceps* have been identified as cordycepin (3′-deoxyadenosine), D-mannitol (cordycepic acid), and polysaccharides, and these are thought to have potential antiaging, whitening, antitumor, anti-inflammatory, immunomodulatory, and/or blood glucose and cholesterol lowering activities [8,9]. However, the active component cordycepin can degrade in vivo due to degradation by enzyme adenosine deaminase and also others environmental factors like sun light, UVB light, and high temperatures [10]. Given the potential medicinal value, we chose *Cordyceps militaris* fungus extract as the aqueous media in our O/W system. *Hippophae rhamnoides* or sea buckthorn is a well-known medicinal plant in Asia, and the oil from its fruit contains ellagic acid, polyunsaturated fatty acids, tocopherols, carotenoids, sterols, amino acids, carbohydrates, and vitamin E. The potential health benefits of sea buckthorn fruit include whitening, antiaging, anticancer, antioxidant, cardiovascular, antidiabetic, anti-inflammation and anti-microbial activity [11,12,13]. Therefore, the objective of this research was to combine the hydrophobic and hydrophilic part of the bioactive compound from *Cordyceps* extracts using surfactant to increase potential efficacy for biomedical applications.

## 2. Results and Discussion

### 2.1. Nanoemulsion Formulation

Many variables are considered in the preparation of NEs for cosmetics or medicinal product formulation. Sea buckthorn oil is often used as the oil phase due to its skin conditioning properties and nongreasy feel. The type of NE (O/W or W/O) depends on the surfactant, water, or oil percentage in each phase. In oil-in-water emulsions, the water content ranges between 50% and 92%, while the surfactant content ranges between 1.5% and 10% [14,15]. Tween 80, a nonionic surfactant, was used as emulsifier for *Cordyceps* nanoemulsion preparations. Different types of nonionic surfactant are suitable for oil in water nanoemulsion preparation, like nonionic polysorbates surfactants (Tweens), but have different effects on size of the droplets. The smallest droplet size can produce by Tween 80 where the hydrophilic lipophilic balance HLB = 15; however, others Tweens like Tween 21 (HLB = 7.6) produce larger droplet size [16]. In Tween 80, the double bond of oleate ester in its hydrophobic chain induce more mobility from organic phase to aqueous phase for faster production of emulsion but the unsaturated surfactant like Tween 21 reduce the mobility of the two phase resulting in poor and unstable nanoemulsion formulation [17]. Therefore, Tween 80 is used as an emulsifier rather than others Tweens. In addition, the surfactant concentration is key factors for droplet size of the nanoemulsion. Surfactant lowers the interfacial tension between the two phase (dispersed and continuous) that leads to smaller particles size as well as the ultrasonic or homogenization time [18]. The higher amount of surfactant can produce the small size when high pressure method is applied due to more reduction in interfacial tension. Besides, the lower concentration of surfactant increase the droplet size due to insufficient amount of surfactant that cannot cover the droplet fully hence coalescence can occurred [19]. Moreover, a high percentage of surfactants may cause skin irritation, so, this study followed 3~10% surfactant concentration [20]

The NEs were prepared using varying proportions of the aqueous COR-Ex, oil, and surfactant, and the stability of the resulting emulsions was assessed. A NE was considered stable only when there was no creaming, flocculation, or phase separation during the observation period [21]. Three different formulations, S1, S2, and S3, were optimized (Table 1). We used oil and surfactant ratio of 1:1, 1:2, and 1:3 where we kept that oil percentage same because high amount of oil content has tendency to form bigger droplet due to higher viscosity and also it encourages the agglomeration that form bigger particles [22]. Among the three formulations, the S2 and S3 formulations showed a little creaming after 7 days of observation. These observations may be due to higher surfactant concentrations that made diffusion layer, which tends to cause excessive coverage of particles by surfactants and finally agglomerates the formulation by lowering the zeta-potential value [23]. Only the S1 formulation did not show any modification and was chosen as the most stable NE. We also used high-molecular-weight chitosan as a polymer because it can reduce instability and aggregation due to high shear thinning (pseudo plastic) with a high viscosity and a low shear rate, which is mostly used to establish a stable continuous phase [24]. So finally, 88% water extract, 0.1% chitosan, 6% oil, and 6% Tween 80 was used for nanoemulsion preparation.

### 2.2. Effect of Storage Time and Conditions

The prepared NEs were stored at a range of temperatures (4 °C, 25 °C, and 60 °C) for 3 months. In terms of physical appearance, the emulsions stored at 4 or 25 °C did not show any color changes and phase separation. After storage at 60 °C, creaming of the NE was evident, but phase separation did not occur; the size of the NE droplets increased twice than the initial value (Table 2) possibly due to the use of a nonionic surfactant that may be thermally sensitive [25]. We also assessed the size of the NE droplets in a different condition where at 4 °C storage conditions, we could not find any significant difference in droplet size. The smaller the droplet size, the better the stability of the NE. The pH of the *Cordyceps* NEs was between 5 and 6 and also had a stable zeta potential of −26. The ideal pH for NEs is 4.5−6.0 to ensure good skin absorption and stability of the formulation [26]. We found that after storage at 60 °C, the pH of the NEs was slightly reduced, but still within the acceptable range; this reduction may have been due to degradation of free fatty acids in the oil phase [27].

The PDI value indicates the monodispersity of the nanoformulation. Generally, PDI values less than 0.250 are consistent with monodispersed emulsions [28]. In this study, PDI values below 0.2 for the NEs demonstrated the uniformity of all the formulations. A zeta potential value of ±20 is considered sufficiently stable to make a protective energy barrier between the droplet due to coalescence, and also a negative zeta potential value was found due to using nonionic surfactant in the formulation [29,30]. At high zeta potential value, there is ability of resisting coalescence, and at low zeta potential value, the attraction exceeds repulsion and breakdowns the dispersion and flocculates it [31]. So, high zeta potential gives stable nanoemulsion and low zeta potential value tends nanoemulsion to coagulate. In this study, the zeta potential for the *Cordyceps* formulations was around −26, which was indicative of a stable formulation. Overall, storage at 4 °C was considered ideal for the *Cordyceps* NE formulations.

### 2.3. FT-IR Analysis

**Figure 1:**
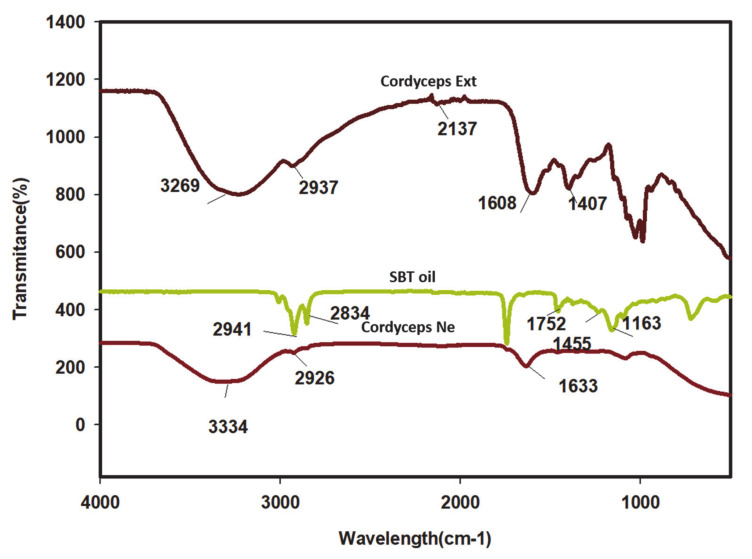
FT-IR analysis for *Cordyceps* nanoemulsion with *Cordyceps* extract (COR-Ex) and sea buckthorn oil.

The FT-IR spectra of the *Cordyceps* NE, COR-Ex, and sea buckthorn oil are shown in Figure 1. The *Cordyceps* extract exhibited absorption bands at 3269, 2937, 2137, 1608, and 1407 cm^−1^, corresponding to –OH broad (phenolic OH), (C-H) stretching, and alkyne (C=O) stretch (amide bond) major functional groups, respectively.

Sea buckthorn oil exhibited typical absorption bands in the hydrophobic region at 3009, 2936, and 2834, which were associated with antisymmetric CH_3_ and CH_2_ scissoring and antisymmetric CH_2_ functional groups, respectively. An additional band was evident at 1752 representing C=O stretching of the ester bond. The COR-NE displayed several strong absorption bands at 3334, 2926, 1636 cm^−1^ associated with –OH stretch (–OH phenolic group). The –CH_2_ stretching (alkane), C=O stretch (amide), and absorption bands at 1458, 1354, 1250, and 1058 cm^−1^ were associated with amides I (N-H bending), ester carbonyl (C-O-C stretch), and polysaccharides, respectively. The functional group at 2926 cm^−1^ represents the hydrophobic interaction between the extract and oil as part of forming the NE and the –OH bond present in the NE, which is also indicated in the COR-Ex.

### 2.4. FE-TEM Analysis

**Figure 2:**
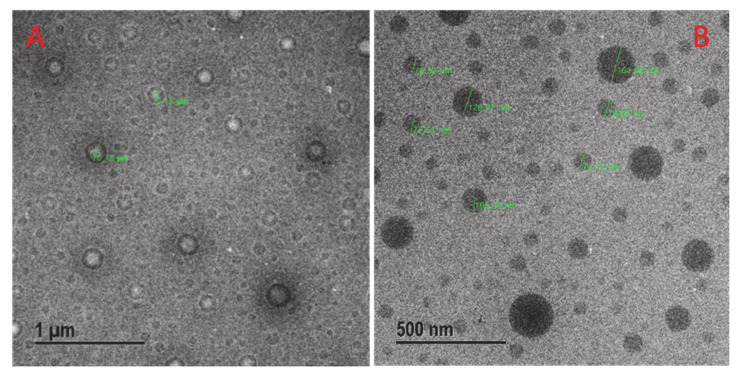
FE-TEM images indicated the spherical shape of *Cordyceps* nanoemulsion at (**A**) 1 μm and (**B**) 500 nm.

The morphology of the *Cordyceps* O/W NE was characterized by FE-TEM, and the droplet size was found to be similar to the diameters (80~100) nm measured by the dynamic light scattering instrument. The TEM images also indicated that droplets were well distributed and spherical in shape, as shown in Figure 2.

### 2.5. In Vitro Cytotoxicity of Cordyceps NE

**Figure 3:**
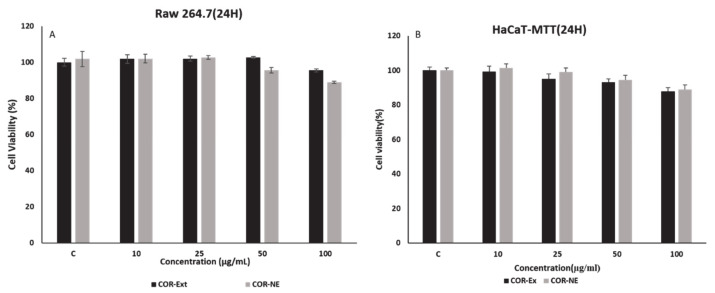
Cell cytotoxicity analysis of *Cordyceps* nanoemulsion using MTT assay on (**A**) macrophage RAW 264.7 cell line and (**B**) human keratinocyte (HaCaT) cell line.

The cytotoxicity of the COR-NE was analyzed using murine macrophage (RAW 264.7) and human keratinocyte (HaCaT) cell lines. We had treated the normal cell lines with COR-NE samples to assess the cytotoxic effects on normal cells to ensure their safety in human applications. The cytotoxicity of the nanoemulsion (10~100 µL concentrations for 48 h) was assessed using the (3-(4, 5-dimethyl-2-thiazolyl)-2, 5-diphenyl-2 H tetrazolium bromide (MTT assay). The COR-NE was nontoxic in both cell lines. Almost 80% of the cells survived exposure to the COR-NE at up to 100 µL/mL, as shown in Figure 3.

### 2.6. Antioxidant Activity of Cordyceps Nanoemulsion

The DPPH assay is based upon inhibition of free radical formation due to the reaction of the stable DPPH radical with an anti-oxidant compound that donates hydrogen to reduce the DPPH. The color changes from violet to yellow due to inhibition of DPPH free radicals is measured at 517 nm. Mostly the phenolic compound is responsible for antioxidant activity that contains (–OH) free radicals [32]. The fruiting body of the cultivated *Cordyceps* was screened using the DPPH assay in order to identify bioactive molecules for medicinal applications.

**Figure 4:**
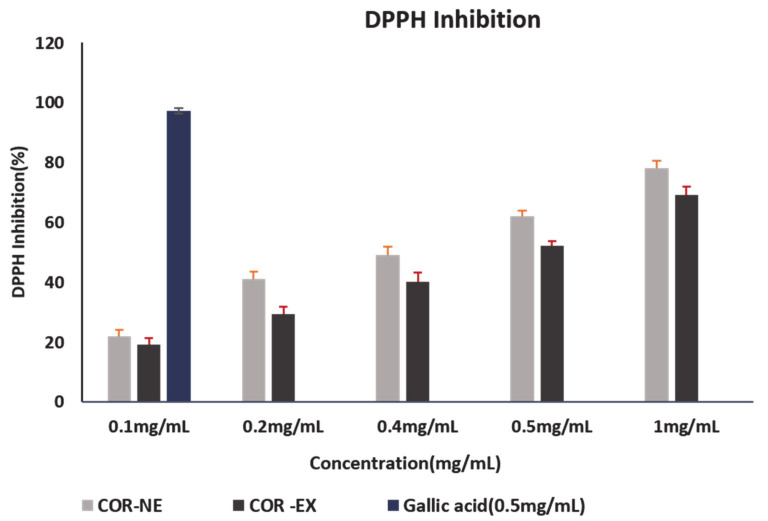
Antioxidant properties of *Cordyceps* nanoemulsion compared with commercial antioxidant agent Gallic acid using DPPH inhibition assay.

Both hydrophilic (free sugars, organic acids, and phenolic acids) and lipophilic (fatty acids and tocopherols) compounds have antioxidant, antimicrobial, and antiproliferative properties [33]. The composition of hydrophilic (phenolic acid and free sugar) and lipophilic (fatty acid and tocopherols) components responsible for antioxidant activity is virtually all related to the polysaccharide extracts of the mushroom. The antioxidant activity of the COR-NE and extract were evaluated, and the NE showed greater antioxidant activity than the free extract. The total antioxidant capacity of the COR-NE and extract as assessed in the DPPH radical scavenging assay were about 78.01% and 69.3%, respectively. The total antioxidant activity of the positive control ascorbic acid (5 mg/mL) was about 97.23% as shown in Figure 4. The antioxidant capacity of the NE was greater than that of the non-capsulated extract, likely because nano encapsulation not only prevented degradation of the COR-Ex but also preserved its antioxidant activity. The IC_50_ value of the COR-Ex and NE as determined by linear regression were about 3.94 ± 0.14 and 2.96 ± 0.10 mg/mL, respectively. Therefore, we postulated that the antioxidant activity of the COR-NE was due to the higher amount of encapsulated *Cordyceps* extract. The antioxidant activities of NEs of *Zingiber officinale* and *Curcuma longa* have been reported [34,35] but this is the first time a COR-NE has been reported to be a better source of antioxidants than an aqueous extract of *Cordyceps militaris* fungus.

### 2.7. Antimicrobial Activity of Cordyceps Nanoemulsion

**Figure 5:**
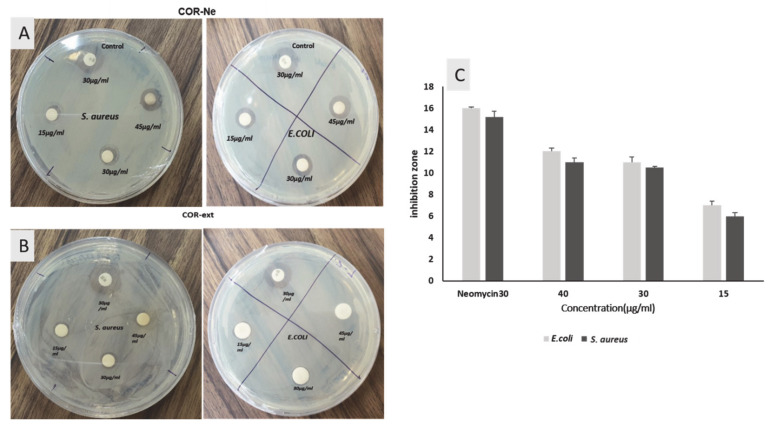
Antibacterial efficacy analysis using disk diffusion method against pathogenic bacteria *E. coli* and *S. aureus:* (**A**) *Cordyceps* nanoemulsion (**B**) COR-Ex, (**C**) bar chart for calculating inhibition zone of *Cordyceps* nanoemulsion against *E. coli* and *S. aureus* compared with commercial antibiotics *Neomycin* 30.

The COR-NE was assessed in a disk diffusion assay with pathogenic Gram-negative *E. coli* and Gram-positive *S. aureus* bacteria, and the results were compared with those produced by commercial *neomycin*-impregnated disks. The zone of inhibition by COR-NE in *E. coli* and *S. aureus* cultures was 11 and 12 mm, respectively, which was similar to the *neomycin 30* disk (shown in Figure 5). The mechanism involved in the inhibition of bacterial growth is the transformation of membrane permeability, and the destruction of protein and nucleic acid structure that inhibit nucleic acid synthesis and permanently damage the cell wall of the bacteria. Overall, this causes cell death with an increase in structural lesions and induces the release of electrolyte, protein, and other cell contents [36].

COR-Ex have previously been shown to inhibit both Gram-positive and Gram-negative bacteria, and Hu et al. isolated a CSAP protein from the mycelia of *Cordyceps* species that showed good antibacterial efficacy [33]. Several bioactive compounds, including cordycepic acid, adenosine, polysaccharides, unsaturated fatty acid, and other nutrients, have been isolated from *Cordyceps* [37], and the polysaccharides have be reported to increase the antibacterial efficacy of the plant [38]. The main advantages of nanoemulsion formulations are the increased stability and solubility of the encapsulated compound and increased efficacy of antibacterial compounds [39]. Our results suggest that increased inhibition of Gram-negative rather than Gram-positive bacteria after formulating the COR-Ex as a nanoemulsion may have been due to differences in the bacterial cell wall/membrane, given the Gram-negative bacterial cell wall renders the outer surface highly hydrophilic. In contrast, teichoic acids in the cell wall of Gram-positive bacteria may facilitate penetration by hydrophobic compounds [40]. In addition, the use of nonionic surfactants can reduce the surface and interfacial tension that are needed to increase the absorption and uptake of the preserved component by microbial cells, which destroy the cells at a faster rate [41].

### 2.8. In Vitro Anti-Inflammatory Activity of Cordyceps NE

**Figure 6:**
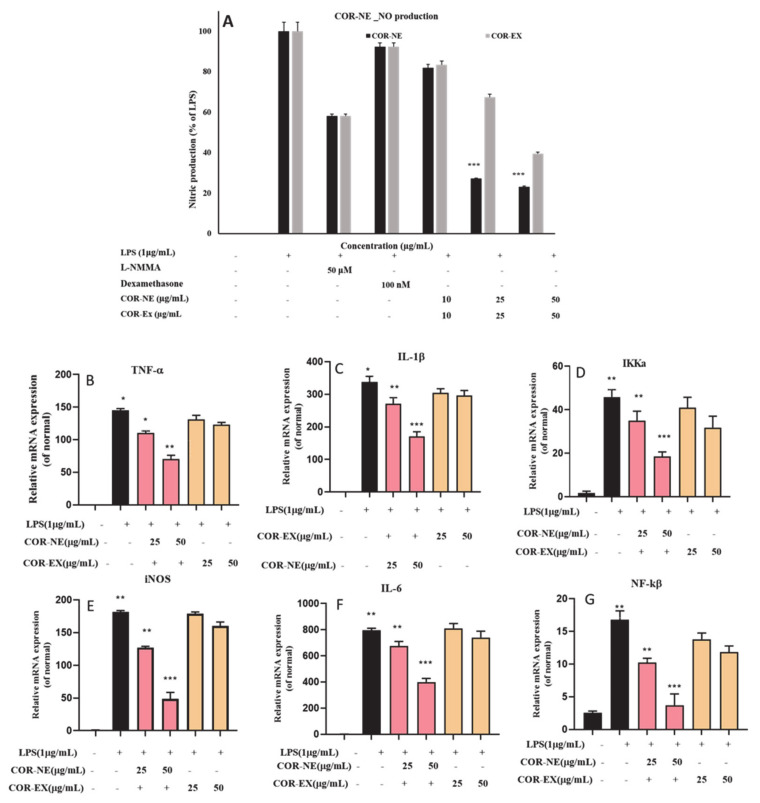
Effect of *Cordyceps* nanoemulsion on: (**A**) NO production and pro-inflammatory cytokines (**B**) TNF-α, (**C**) IL-1β, (**D**) IKKa, (**E**) iNOS, (**F**) IL-6, and (**G**) NF-kß mRNA expression in LPS-induced RAW 264.7 cells. Each value is expressed as the mean ± standard error of three independent experiments. * *p* < 0.05, ** *p* < 0.01, *** *p* < 0.001 compared with control.

The influence of various inflammatory mediators, including NO production and signaling mechanisms, is correlated with many diseases linked to inflammation. The current study assessed effects of the synthesized NE on the viability of the RAW 264.6 cell line and in NO release assays. The results highlighted that neither the *Cordyceps* NE nor the free extract had any significant toxicity to human cell line at concentration up to 100 µg/mL. Nevertheless, the NE significantly inhibited LPS-induced NO production, as shown in Figure 6. This inhibition of NO production may have been due to greater entrapment of cordycepin [42] from the COR-Ex and the synergistic effect of sea buckthorn oil. Therefore, in this study, we successfully developed a *Cordyceps* O/W NE as a potential candidate for the future treatment of inflammatory diseases. Pro-inflammatory cytokines have a significant impact on the inflammatory response. The pro-inflammatory cytokines in LPS-induced macrophages were measured using an ELISA. To assess the anti-inflammatory effects of the COR-NE, the gene expression of cytokines was measured in an LPS-induced RAW 264.7 cell model by qPCR analysis. The RAW cells were incubated with 25 and 50 µg/mL or without the LPS; however, at a concentration of 1 µg/mL, LPS was used as inducer for 24 h. Figure 6 presents the results of the q-PCR analyses indicating that the COR-NE significantly suppressed inflammation by reducing the expression of mRNA for IL-1β, TNF-α, IL-6, iNOS, NF-κB, and IKKα. These results suggest that the COR-NE may suppress inflammation by nitric oxide inhibition and downregulation of the mRNA expression.

## 3. Materials and Methods

### 3.1. Materials

The COR-Ex was prepared from *Cordyceps* mushrooms cultured in our laboratory. Sea buckthorn oil (fruit oil) was obtained from Inner Mongolia, China. Lactic acid, NaOH, and HCl were purchased from Dae-Jung Chemical Co., Siheung-si, Korea. An ultrasonic probe (3000, Newtown, CT, USA, probe diameter 3 mm), Tween 80, and 2,2-diphenyl-1-picrylhydrazyl (DPPH) were purchased from Sigma-Aldrich (St Louis, MO, USA). Fetal bovine serum (FBS) and *penicillin-streptomycin* solution were purchased from Gen DEPOT (Barker, TX, USA). High-glucose Dulbecco’s Modified Eagle’s Medium (DMEM) with pyruvate was purchased from Gibco (Waltham, MA, USA). MTT solution was bought from Life Technologies (Eugene, OR, USA). Chitosan from crab shell (85%, deacylated) was collected from Sigma (Cat. No. C36462). Neomycin (30 μg/disk) antibiotic disks were obtained from Becton Dickinson, and Company (Seoul, Korea). The *Staphylococcus aureus* (ATCC6538) and *Escherichia coli* (BL-21) strains were cultured at 37 °C on nutrient agar media purchased from Difco, MB Cell, Seoul, Korea, and stored at −70 °C in glycerol stock vials for future experiments. Standard *Neomycin 30* (NEO30) discs were purchased from Oxoid Ltd. (Thermo Fisher Scientific, Gangnam-gu, Seoul, South Korea).

### 3.2. Methods

#### 3.2.1. Extract Preparation

A total of 100 g of dried *Cordyceps* powder was placed in 2 L of distilled water and sonicated for almost 2 h at 20−28 °C. The COR-Ex was centrifuged at 8000 rpm for approximately 20 min and the supernatant was collected. The extract was analyzed by HPLC to calculate the percentage of adenosine and cordycepin compared with the standard sample using different analyses (as shown in the Supplementary Materials): with (MeOH) 13%, H_2_O 87%, a flow time of 1.0 mL/min; a stop time of 25 min; and an injection of 1 and 10 µL. The prepared extract was used for further experiments.

#### 3.2.2. Nanoemulsion Preparation Method 

The NE was prepared as described previously with minor modifications [43]. Optimization of the COR-NE was performed using different extracts, oils, surfactants, and co-surfactants. A total of three samples were prepared and optimized, and their stability was assessed. The NE was prepared using 10 g of *Cordyceps* (extracted using 100 g/2 L water) sea buckthorn oil, Tween 80 (nonionic surfactant), and chitosan (from crab shell) as a co-surfactant added at 1% of the total volume. A total of three samples were prepared for optimized stability, namely, S1, S2, and S3. 

To prepare the coarse emulsion, the water extract was poured into a conical flask and stirred continuously at 500 rpm at room temperature using a magnetic stirrer while adding the oil, surfactant, and co-surfactant. The preparation was then subjected to an ultrasonic probe (VCX 130 ultrasonic processor, from Sonics and materials Inc., (Newtown, CT, USA) for NE formation. The probe frequency was set to 20 kHz and 130 W, the amplitude was set to 50%, and the probe diameter was 3 mm (titanium alloy). The whole process was completed within 15 min using a 10 s pulse and 1 s off cycle. Three preparations were prepared using the same conditions. We performed the whole experiment using an ice bath to avoid any thermal damage and kept the NE formulation at 4 °C and the flasks were wrapped with foil for further experiments. Finally, the best optimized sample was used for characterization and further experiments.

#### 3.2.3. Characterization Process

Several analytical methods were used to determine the size, shape, stability, and morphology of the NE. The morphology and size were confirmed using field-emission transmission electron microscopy (FE-TEM; JEM-2100 F, JEOL, Suwon, Korea). One drop of the NE was deposited onto a carbon grid and dried in a 60 °C oven for 10 min and placed for capturing images. The particle size and zeta potential of the NE were analyzed using dynamic light scattering (DLS; Otsuka Electronics, Shiga, Japan). The NE preparations were diluted 10-fold with distilled water for evaluation of the Z-average diameter, zeta potential, and the polydispersity index (PDI). The temperature was maintained at 25 °C and as a reference condition, a dispersive medium of pure water with a refractive index of 1.3328, a dielectric constant of 78.3, and viscosity of 0.8878 was used. Fourier-transform infrared spectroscopy (FT-IR; Perkin Elmer Spectrum One FT-IR spectrometer, Boston, MA) was performed in the range of 450–4000 cm^−1^.

#### 3.2.4. Determination of the Stability of the Nanoemulsions

The COR-NE was optimized in various conditions such as the temperatures and pH (3–7). The optimal storage condition was determined after storing the NE at various temperatures (4, 25, and 60 °C) for 3 months. After each treatment, the droplet size and zeta potential were measured to assess the stability of the NEs. 

#### 3.2.5. Antioxidant Activity

The antioxidant activities of the COR-NE and free COR-Ex were determined using a 2,2-diphenyl-1-picrylhydrazyl (DPPH) free radical scavenging assay. Gallic acid was used as a positive control. For the assay, 160 μL of a 0.1 mM methanolic solution of DPPH was added to 40 μL of COR-NE or COR-Ex (2−20 μg/mL). The reaction mixture was incubated at 37 °C for 30 min and the absorbance measured at 517 nm using a Synergy™ 2 multimode reader (Bio-Tek Instruments, Inc., Vinooski, VT, USA). The inhibition percentage was calculated using the following formula:

[Absorbance of sample−Absorbance of Control/absorbance of control] × 100(1)

The absorbance of the sample and the DPPH solution and concentration required to scavenge 50% of the DPPH free radicals (IC50) were calculated.

#### 3.2.6. In Vitro Measurement of NO Production in LPS-Induced RAW 264.7 Cells 

An LPS-induced RAW 264.7 cell line was used to measure production of pro-inflammatory nitric oxide (NO). Cells were seeded into a 96-well plate at 1 × 104 cells/well. The cells were pretreated for 1 h with various concentrations of the prepared *Cordyceps* NE, and the cells were then stimulated with 1 µg/mL LPS (Sigma-Aldrich, St. Louis, MO, USA). After 24 h incubation, the supernatant was collected, and NO production was determined using a colorimetric Griess reaction, for which 100 mL of premixed Griess reagent (Sigma-Aldrich, St. Louis, MO, USA) was added to an equal volume of supernatant and incubated for 10 min and DMSO was added to dissolve the formazan into the colored solution. The absorbance of each sample was measured at 450 nm using a multimode microplate reader.

#### 3.2.7. Reverse Transcription-Polymerase Chain Reaction (RT-PCR)

The RAW 264.7 cells were pretreated with the COR-NE and COR-Ex for 1 h, and then, LPS (1 µg/mL) was added and the cells were incubated for 24 h, as above. Total RNA was collected using a TRIzol Reagent Kit (Invitrogen Company, Carlsbad, CA, USA). Total RNA (2000 ng) was reverse transcribed with reversed transcriptase and an oligo (dt) primer for cDNA (AccuPower RT PreMix, Bioneer Co., Daejeon, South Korea). The reaction was performed at 42 °C for 60 min, followed by heating to 94 °C for 5 min. Then, 1 μL of the cDNA mixture was used for PCR amplification using AccuPower Hot Start PCR PreMix (Bioneer Co., Daejeon, Korea) as described previously. The primer sequence is provided in Table 3. The PCR products were separated using 2% agarose gel electrophoresis and stained with ethidium bromide and, finally, detected by UV illumination. Glyceraldehyde 3-phosphate dehydrogenase (GADPH), a housekeeping gene, was used as the control. All experiments were performed triplicate.

#### 3.2.8. Cytotoxicity Assay

The in vitro cytotoxicity was performed as described previously with minor modifications [44]. The RAW 264.7 and HaCaT (skin keratinocyte) cell lines (both obtained from KCLB, Seoul, Korea) were cultured in Dulbecco’s Modified Eagles Medium (DMEM; Gibco-BRL, Grand Island, NY, USA) with 10% FBS and 1% *penicillin/streptomycin* (WElGENE Inc, Daegu, Korea) and incubated at 37 °C in a humidified atmosphere containing 5% CO_2_ and 95% air. The cells were seeded into 96 well plates at 1 × 10^4^ cells/well. The COR-NE and extract (0, 10, 25, 50, 75, or 100 µg/mL each) was added and the cells incubated for 24 h at 37 °C. After incubation, 20 µL of a 5 mg/mL MTT solution (3-(4, 5-dimethyl-2-thiazolyl)-2, 5-diphenyl-2 H tetrazolium bromide in PBS (Life Technologies, Eugene, OR, USA) was added to each well, and the plates were incubated for an additional 4 h at 37 °C. Viable cells converted the MTT solution to purple-colored formazan, and the insoluble formazan was dissolved by adding 100 µL of DMSO to each well and the absorbance was measured at 570 nm with an enzyme-linked immunosorbent assay (ELISA) reader (Bio-Tek, Instruments, Inc, Winooski, VT, USA).

#### 3.2.9. Antimicrobial Activity

The antimicrobial activity of the COR-NE was assessed using the disk diffusion method. The pathogenic microorganisms *Escherichia coli* and *Staphylococcus aureus* were grown on Muller Hinton agar (MHA) plates. In this assay, 100 µL of log-phase bacterial cultures grown overnight were spread evenly onto the MHA plate. The COR-NE was impregnated onto new paper disks, and standard *Neomycin* antibiotic disks were used as the positive control. The disks were placed onto the surface of MHA plates for a 24-h incubation period at 37 °C. After incubation, the zone of inhibition was measured and compared with control drug.

## 4. Conclusions

A *Cordyceps* oil-in-water NE was prepared from an aqueous COR-Ex using ultrasonication. The COR-NE was characterized using Fourier-transform infrared spectroscopy (FT-IR), dynamic light scattering (DLS), and field-emission transmission electron microscopy (FE-TEM). At initial preparation, DLS analysis indicated that the droplet size was 87.0 ± 2.1 nm, the PDI value was 0.089 ± 0.023, and the zeta potential was −26.20 ± 2. The small droplet size, low PDI, and stable zeta potential highlighted the excellent stability of the NE. The stability of the NE was evaluated under different temperatures and storage conditions. However, after 90 days, stability was checked at different temperatures conditions (4, 25, and 60 °C) where 4 °C storage conditions do not affect the materials’ stability. The NE displayed good antioxidant activity in the DPPH assay (IC50 = 3.94 ± 0.14 mg/mL) and inhibited the growth of *E. coli* and *S. aureus* bacteria. The results of the in vitro cytotoxicity and anti-inflammation assays suggested that the NE was not toxic to RAW 264.7 and HaCaT cells at up to 100 µL/mL. The COR-NE also had strong anti-inflammatory activity in LPS-induced RAW 264.7 cells at 50 µL/mL, as confirmed by inhibition of NO release and qPCR analysis of genes expressions related to inflammation. The COR-NE appeared to suppress the inflammation by reducing the expression of IL-1β, TNF-α, IL-6, iNOS, NF-κB, and IKKα mRNAs expressions. Moreover, *Cordyceps* nanoemulsion can be a better option for future nano drug for biomedical applications.

## Figures and Tables

**Table 1:** Optimization of the *Cordyceps* nanoemulsion preparation process.

Water Extract (*Cordyceps*)		Sea-Buckthorn Oil	Surfactant	Co-surfactant (Chitosan)
S1	88%	6	6	0.1%
S2	82%	6	12	0.1%
S3	78%	6	18	0.1%

**Table 2:** *Cordyceps* nanoemulsion stability test after 90 days observations.

No. of Parameter	*Cordyceps* Nanoemulsion			
	24 h Observation	90 Days Observation		
Temperature	Room Temperature	4 °C	25 °C	60 °C
Particles size (nm)	87.0 ± 2.1	87.1 ± 3	114.5 ± 2	161.8
PDI value	0.089 ± 0.023	0.100 ± 0.030	0.122 ± 0.04	0.106 ± 0.04
Zeta potential (mV)	−26.20 ± 2	−25.94 ± 0.7	−19.81 ± 0.5	−12 ± 1.2
pH value	5.43 ± 0.05	5.4 ± 0.02	5.39 ± 0.04	5.33 ± 0.012

**Table 3:** List of primer.

GAPDH	Forward	5′-ACCACAGTCCATGCCATCAC-3
	Reverse	5′-CCACCACCCTGTTGCTGTAG-3
IL-1β	Forward	5′-TGCAGAGTTCCCCAACTGGTACATC-3′
	Reverse	5′-GTGCTGCCTAATGTCCCCTTGAATC-3′
TNF-α	Forward	5′-AGCCCACGTCGTAGCAAACCACCAA-3′
	Reverse	5′-AACACCCATTCCCTTCACAGAGCAAT-3′
IL-6	Forward	5′-GTTCTCTGGGAAATCGTGGA-3′
	Reverse	5′-TGTACTCCAGGTAGCTATGG-3′
iNOS	Forward	5′-AATGGCAACATCAGGTCGGCCATCACT-3
	Reverse	5′-GCTGTGTGTCACGAAGTCTCGAACTC-3
NFκB	Forward	5′-TATTTCAACCACAGATGGCACTGC-3
	Reverse	5′-CAGATTTTGACCTGAGGGTAAGAC-3
IKKα	Forward	5′-GGCCTGTGATGTCCTGAAGAATT-3
	Reverse	5′-TCGAATCCCAGACCCTATATCACT-3

## Supplementary Materials

The following are available online. Figure S1. The identification of Adenosine and Cordycepin in Cordyceps extract. Figure S2. The Adenosine and Cordycepin percentage in *Cordyceps* extract.
