# Supplementary material for: Cordyceps militaris Fungus Extracts-Mediated Nanoemulsion for Improvement Antioxidant, Antimicrobial, and Anti-Inflammatory Activities
Source: Molecules. 2020 Dec 4;25(23):5733. doi: 10.3390/molecules25235733 (PMC7730259; doi:10.3390/molecules25235733)
Supplement: Supplementary file 1 [file molecules-25-05733-s001.zip › suplementary ppt.pptx]

## Slide 1
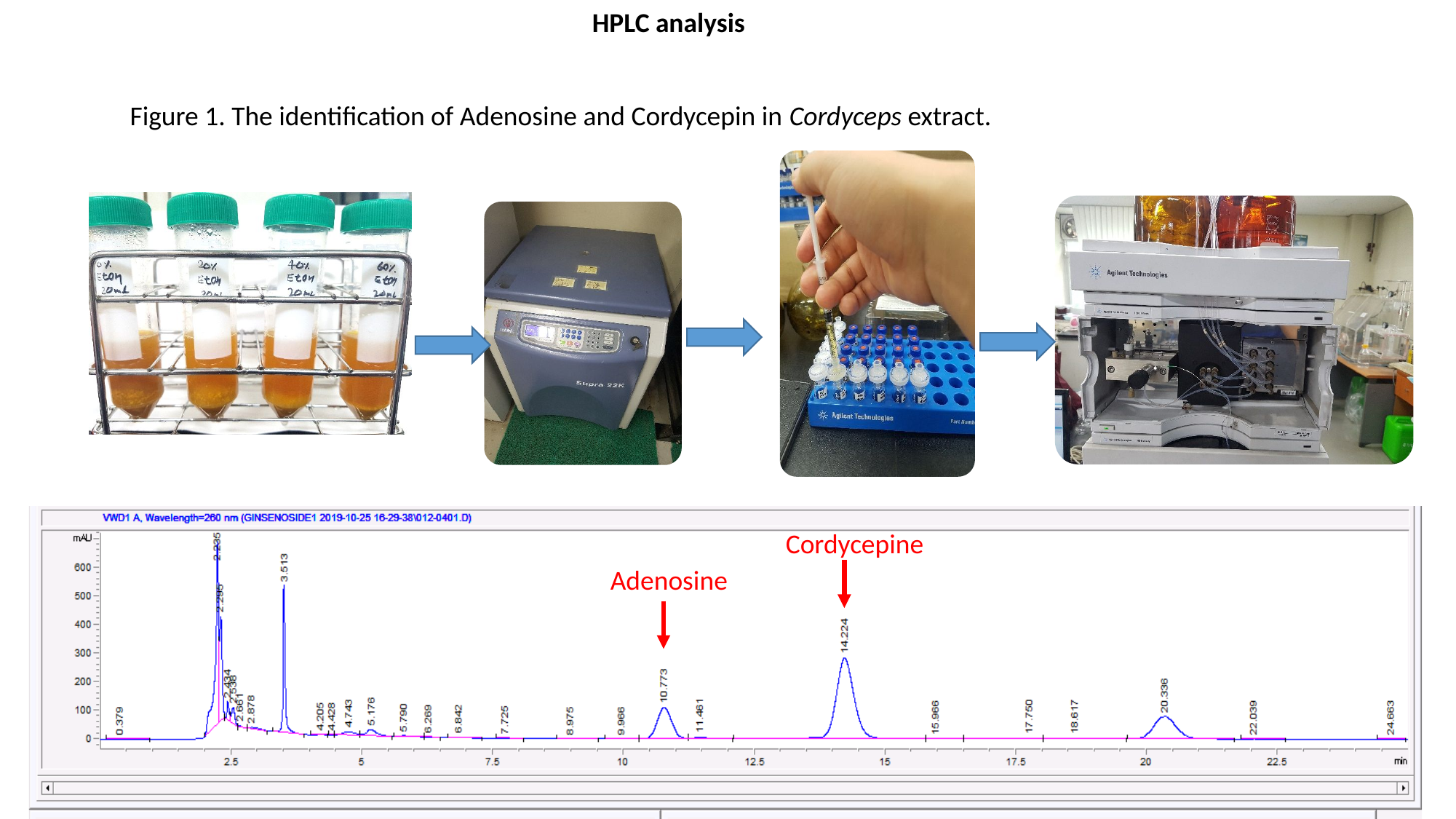

HPLC analysis
Figure 1. The identification of Adenosine and Cordycepin in Cordyceps extract.
Cordycepine
Adenosine

## Slide 2
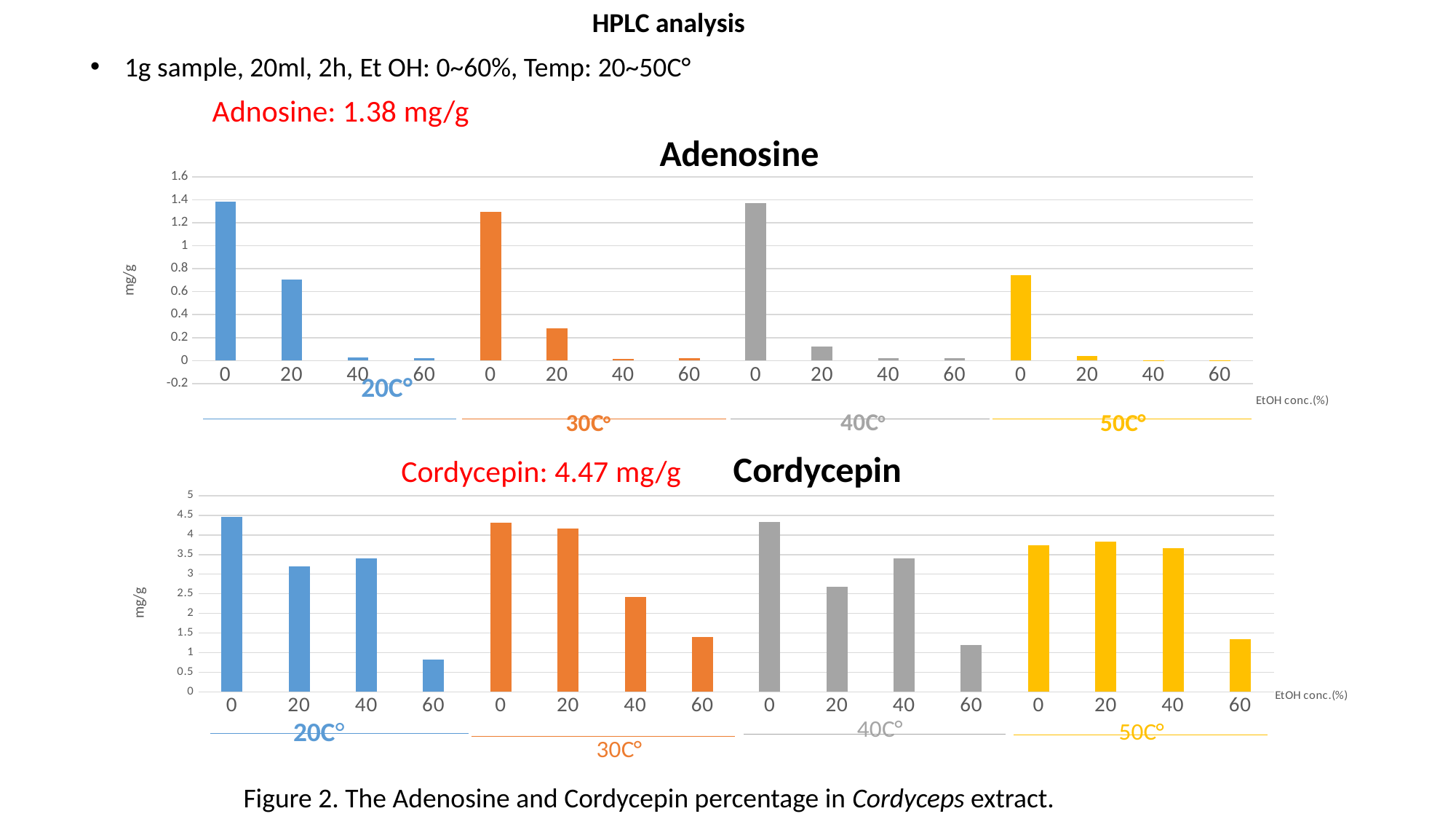

HPLC analysis
1g sample, 20ml, 2h, Et OH: 0~60%, Temp: 20~50C°
Adnosine: 1.38 mg/g
### Chart: Adenosine
| Category | |
|---|---|
| 0 | 1.3819449963400607 |
| 20 | 0.7050653560598139 |
| 40 | 0.024212067342884034 |
| 60 | 0.021388685558925025 |
| 0 | 1.2971041165603543 |
| 20 | 0.2810352399874516 |
| 40 | 0.017449893687476035 |
| 60 | 0.023026944124925924 |
| 0 | 1.3733005681620134 |
| 20 | 0.12292586008574714 |
| 40 | 0.02372407542960717 |
| 60 | 0.019192721949179127 |
| 0 | 0.7452201192094531 |
| 20 | 0.03909582069782844 |
| 40 | 0.004657534246575341 |
| 60 | -0.005799435323643207 |
### Chart: Cordycepin
| Category | |
|---|---|
| 0 | 4.469393925222719 |
| 20 | 3.1943921715341266 |
| 40 | 3.4065183435826683 |
| 60 | 0.8162501461407159 |
| 0 | 4.318483176280777 |
| 20 | 4.157424416021699 |
| 40 | 2.4111246989501245 |
| 60 | 1.3944062010428602 |
| 0 | 4.33761007318727 |
| 20 | 2.6715591460705683 |
| 40 | 3.394125610868192 |
| 60 | 1.1930827507190125 |
| 0 | 3.7458453948137587 |
| 20 | 3.8218853321486193 |
| 40 | 3.66765426613978 |
| 60 | 1.3427308438749503 |Cordycepin: 4.47 mg/g
Figure 2. The Adenosine and Cordycepin percentage in Cordyceps extract.
